# Comparison of bladder carcinogenesis biomarkers in the urine of traditional cigarette users and e-cigarette users

**DOI:** 10.3389/fpubh.2024.1385628

**Published:** 2024-04-23

**Authors:** Lida Feng, Guixiao Huang, Lei Peng, Rui Liang, Dashi Deng, Shaohua Zhang, Guangzhi Li, Song Wu

**Affiliations:** ^1^School of Medicine, Anhui University of Science and Technology, Huainan, Anhui, China; ^2^Institute of Urology, The Affiliated Luohu Hospital of Shenzhen University, Shenzhen University, Shenzhen, China; ^3^Department of Motor Robotics Institute (MRI), South China Hospital, Heath Science Center, Shenzhen University, Shenzhen, China; ^4^Lanzhou University Second Hospital, Lanzhou, China; ^5^Department of Urology, The First Affiliated Hospital of Soochow University, Suzhou, China

**Keywords:** traditional tobacco, e-cigarette, bladder cancer, polycyclic aromatic hydrocarbons, urine biomarker

## Abstract

**Background:**

During the use of electronic cigarettes (e-cigarettes), users are still exposed to carcinogens similar to those found in tobacco products. Since these carcinogens are metabolized and excreted in urine, they may have carcinogenic effects on the bladder urinary tract epithelium. This meta-analysis aimed to compare bladder cancer carcinogens in the urine of tobacco users and e-cigarette users using a large number of samples.

**Methods:**

A systematic meta-analysis was performed using data obtained from several scientific databases (up to November 2023). This cumulative analysis was performed following the Preferred Reporting Items for Systematic Evaluation and Meta-Analysis (PRISMA) and Assessing the Methodological Quality of Systematic Evaluations (AMSTAR) guidelines, according to a protocol registered with PROSPERO. This study was registered on PROSPERO and obtained the unique number: CRD42023455600.

**Results:**

The analysis included 10 high-quality studies that considered polycyclic aromatic hydrocarbons (PAHs), volatile organic compounds (VOCs) and tobacco-specific nitrosamines (TSNAs). Statistical indicators show that there is a difference between the tobacco user group and the e-cigarette user group in terms of 1-Hydroxynaphthalene (1-NAP) [weighted mean difference (WMD)10.14, 95% confidence interval (CI) (8.41 to 11.88), *p* < 0.05], 1-Hydroxyphenanthrene (1-PHE) [WMD 0.08, 95% CI (−0.14 to 0.31), *p* > 0.05], 1-Hydroxypyrene (1-PYR) [WMD 0.16, 95% CI (0.12 to 0.20), *p* < 0.05], 2-Hydroxyfluorene (2-FLU) [WMD 0.69, 95% CI (0.58 to 0.80), *p* < 0.05], 2-Hydroxynaphthalene (2-NAP) [WMD 7.48, 95% CI (4.15 to 10.80), *p* < 0.05], 3-Hydroxyfluorene (3-FLU) [WMD 0.57, 95% CI (0.48 to 0.66), *p* < 0.05], 2-Carbamoylethylmercapturic acid (AAMA) [WMD 66.47, 95% CI (27.49 to 105.46), *p* < 0.05], 4-Hydroxy-2-buten-1-yl-mercapturic acid (MHBMA) [WMD 287.79, 95% CI (−54.47 to 630.04), *p* > 0.05], 4-(Methylnitrosamino)-1-(3-pyridyl)-1-butanone (NNAL) [WMD 189.37, 95% CI (78.45 to 300.29), *p* < 0.05], or N0-nitrosonornicotine (NNN) [WMD 11.66, 95% CI (7.32 to 16.00), *p* < 0.05].

**Conclusion:**

Urinary bladder cancer markers were significantly higher in traditional tobacco users than in e-cigarette users.

**Systematic review registration**: PROSPERO (CRD42023455600: https://www.crd.york.ac.uk/PROSPERO/).

## Introduction

As an alternative to conventional tobacco products, e-cigarettes are experiencing a swift surge in popularity among the youth demographic in Europe, America, and China, attributable to their diverse flavors, stylish esthetics, and user-friendly design (1, 2). There is robust evidence indicating that e-cigarettes containing nicotine exhibit a higher efficacy in promoting smoking cessation rates when contrasted with nicotine-free counterparts (3, 4). In contrast to tobacco smoke, which comprises thousands of chemicals, e-cigarettes are devoid of tobacco, obviate the need for combustion, and eschew the production of sidestream smoke. Operated by batteries, these devices deliver an aerosol—commonly referred to as ‘vapor’—to the user, originating from an e-liquid with a well-defined chemical composition. The e-liquids typically consist of varying proportions of glycerin and propylene glycol, serving as the aerosol’s base, and may incorporate nicotine along with a spectrum of flavors (5). Although the vapor produced by e-cigarettes contains fewer chemicals than tobacco smoke, some of the same carcinogens that are found in tobacco smoke have been detected in e-cigarette vapor. It was also observed that chemicals contained in e-cigarette aerosols reduced the activity of DNA repair proteins in mouse lung, bladder, and heart tissues. Consequently, chronic exposure has been shown to prompt adenocarcinoma formation in the lungs and induce uroepithelial hyperplasia and carcinoma in the bladder of mice (6). Therefore, a meta-analysis was performed on biomarkers of bladder carcinogenesis in the urine of traditional cigarette users versus e-cigarette users. This meta-analysis was structured to encompass presently published markers associated with urinary tract carcinogenesis related to bladder cancer, such as PAHs, VOCs, and TSNs found in urine. The study aims to discern the comparative impact of traditional tobacco use versus e-cigarette use on bladder cancer, with the overarching goal of promoting e-cigarettes as a potent tool for smoking cessation, ultimately leading to complete abstinence. Additionally, it seeks to raise awareness among youth and relevant authorities about the hazards associated with e-cigarette use among this demographic.

## Methods

### Protocol

The authors conducted a systematic literature review following the Preferred Reporting Items for PRISMA guidelines (7). This cumulative analysis was performed following the Preferred Reporting Items for Systematic Evaluation and Meta-Analysis (PRISMA) and Assessing the Methodological Quality of Systematic Evaluations (AMSTAR) guidelines (8), according to a protocol registered with PROSPERO (CRD42023455600).

### Literature search and eligibility criteria

Database Search Two investigators (Lida Feng. And Lei Peng.) conducted a systematic search of PubMed, Embase, and Web of Science for eligible studies up to September 2023, using the following keywords: “cancer biomarkers,” “cancer biomarkers urine,” “urinary bladder neoplasms,” “bladder cancer,” “cigarette smoking,” “e-cigarette vapor,” “1-Hydroxynaphthalene” OR “1-NAP,” “1-2-Hydroxynaphthalene” OR “2-NAP,” “1-Hydroxyphenanthrene” OR “1-PHE,” “1-Hydroxypyrene” OR “1-PYR,” “2-Hydroxyfluorene” OR “2-FLU,” “3-Hydroxyfluorene” OR “3-FLU,” “2-Carbamoylethylmercapturic acid” OR “AAMA,” “4-Hydroxy-2-buten-1-yl-mercapturic acid” OR “MHBMA,” “N0-nitrosonornicotine” OR “NNN,” and “4-(Methylnitrosamino)-1-(3-pyridyl)-1-butanone” OR “NNAL.” Inclusion criteria for the article were as follows: (1) urinary biomarkers involving traditional tobacco and e-cigarette users; (2) inclusion of at least one urinary biomarker and provision of the method of extraction of the urinary marker and the amount contained; (3) inclusion of e-cigarette-only or traditional tobacco-only users in each of the individual studies; (4) retrospective or prospective studies; and (5) studies rated as high quality by the Study Quality Rating System. Exclusion criteria included (1) studies on secondary exposure to traditional tobacco, secondary exposure to e-cigarette aerosols, and nontraditional tobacco users; (2) studies that included only traditional tobacco users as well as e-cigarette users; (3) studies with inaccessible data, no index data, and incomplete experimental data and (4) studies with data from animal experiments, theoretical experiments, computerized experiments, and non-*in vivo* experiments.

### Quality evaluation

The Newcastle–Ottawa Scale (NOS) was used to evaluate the quality of the included studies (9). The quality of the included studies was assessed by two authors according to, selection of hypothesis-free cohort (SNEC); representativeness of cohort (REC); demonstration of absence of outcome of interest at the start of the study (DO); ascertainment of exposure (AE); study control for most important factors (SC); adequacy of cohort follow-up (≥80%) (AFU); and follow-up long enough for the outcome to occur (FU); to perform outcome assessment. The research achieving scores higher than six stars was categorized as quality work in accordance with the scale evaluation areas of exposure, comparability, and selection.

### Data extraction

The included studies were analyzed using standard Excel spreadsheets for independent data extraction and entry. The extracted data should include: author, year, study design, intervention, region, age, sample size, male/female, racist, 1-NAP, 2-NAP, 1-PHE, 1-PYR, 2-FLU, 3-FLU, AAMA, MHBMA, NNN, and NNAL.

### Statistical analysis

Stata 16 (StataCorp LLC, University City, TX, USA) was used for statistical analysis. Weighted mean difference (WMD) were used to represent continuous variables, with 95% CI and *p*-values for all outcome indicators. *Q*-tests and chi-square tests (*I*^2^) were used to verify heterogeneity among the included studies. Combining the results using a random-effects model resulted in a more conservative comparison of those who smoked versus those who used e-cigarettes. In addition to subgroup analyses, we performed sensitivity analyses to try to explain the sources of heterogeneity. The Begg test was used to assess and indicate publication bias between studies.

## Results

### Description of studies

**Figure 1:**
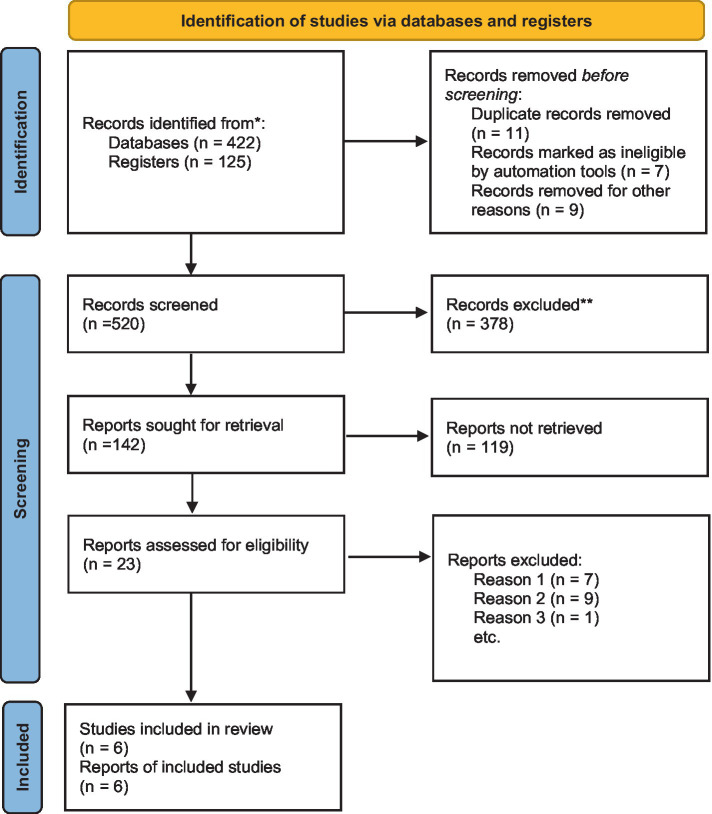
Flow diagram of studies selection process.

A total of 422 publications were retrieved from the five databases, of which 125 were manually retrieved from the references of related studies. Of a total of 547 studies, 541 were excluded for the following reasons: duplications (10), records marked as ineligible by automation tools (*n* = 7), records removed for other reasons (*n* = 9), irrelevant topics (378), reports not retrieved (119), and reports excluded (11). Finally, data from 16,876 patients were included in six retrospective study meta-analysis (Figure 1).

Baseline data extracted from each of the included studies are presented in Table 1, including Author, year of publication, Study design, intervention, region, age, sample size, male/female, and racist.

**Table 1:** Baseline data for studies included in the meta-analysis.

Author, year	Study design	Intervention	Region	Age	Sample Size	Male/Female	Racist
Dai et al. (2022) (12)	Retrospective	Use only cigarettes/Use only E-cigarettes/Both	USA	≥18	3,211	1,462 /1785	68.3%White, 13.2% Black, and 11.8%Hispanic
Maciej L et al. (2018) (11)	Retrospective	Neither/Use only cigarettes/Use only E-cigarettes/Both	USA	35–54	5,105	2042/3063	non-Hispanic white (61%; 95% CI, 58–64%)
Maciej L et al. (2017) (13)	Retrospective	Use only cigarettes/Use only E-cigarettes	USA	20–52	20	8/12	White
Wang et al. (2019) (14)	Retrospective	Neither/Use only cigarettes/Use only E-cigarettes/Both	USA	12–24	8,327	N/A	N/A
Gerhard et al. (2022) (15)	Retrospective	Neither/Use only cigarettes/Use only E-cigarettes	N/A	N/A	60	30/30	N/A
Elaine K et al. (2018) (16)	Retrospective	Use only cigarettes/Use only E-cigarettes	USA	21–60	253	N/A	N/A

USA, United States of America; NA, Not Available.

### Quality assessment

The scores of the included studies based on the NOS Research Quality Score Scale are shown in Table 2.

**Table 2:** Quality score of included studies based on the NOS scale.

Study	Quality indicators from the NOS								Total scores
	Selection				Comparability	Outcome			
	SNEC	REC	DO	AE	SC	AFU	FU	AO	
Dai et al. (2022) (12)	☆	☆	☆	☆	☆☆	☆	☆		8
Maciej L et al. (2018) (11)	☆	☆	☆	☆	☆	☆	☆		7
Maciej L et al. (2017) (13)	☆	☆	☆	☆	☆	☆	☆	☆	8
Wang et al. (2019) (14)	☆	☆	☆	☆	☆☆	☆	☆		8
Gerhard et al. (2022) (15)	☆	☆	☆	☆	☆	☆		☆	7
Elaine K et al. (2018) (16)	☆	☆	☆	☆	☆	☆			6

SNEC, selection of the none posed cohort; REC, representativeness of the cohort; DO, demonstration that outcome of interest was not present at start of study; AE, ascertainment of exposure; SC, study controls most important factors; AFU, adequacy of follow-up of cohort (≥ 80%); FU, follow-up long enough for outcomes to occur; AO, assessment of outcome.

### Urinary biomarkers

Results of each included study are summarized in tabular and narrative form. Urine biomarkers and parent compounds were categorized according to the International Agency for Research on Cancer (IARC) monograph on human carcinogenic risk assessment (17). These compounds were then cross-referenced using the Health and Environment, Toxicology and Disease Collaboration (HEDTC) database to identify associations with bladder cancer and grouped according to the strength of the evidence, and those with some association with bladder cancer were selected for analysis (Table 3) (10).

**Table 3:** Toxicants, carcinogens, and urinary biomarkers detected in the urine e-cigarette users, carcinogenic risk, and link to bladder cancer.

Chemical class	Urinary biomarker(s) (abbreviation)	IARC monographs on the evaluation of carcinogenic risks to humans—classification group	Link to bladder cancer—strength of evidence
Polycyclic aromatic hydrocarbons			
	1-Hydroxynaphthalene (1-NAP)	2B	strong
	2-Hydroxynaphthalene (2-NAP)	2B	
	1-Hydroxyphenanthrene (1-PHE)	3	strong
	1-Hydroxypyrene (1-PYR)	3	strong
	2-Hydroxyfluorene (2-FLU)	3	strong
	3-Hydroxyfluorene (3-FLU)	3	
Volatile organic compounds			
	2-Carbamoylethylmercapturic acid (AAMA)	2A	Strong, cancer not otherwise specified
	4-Hydroxy-2-buten-1-yl-mercapturic acid (MHBMA)	1	Strong, cancer not otherwise specified
Tobacco-specific nitrosamines			
	N0-nitrosonornicotine (NNN)	1	limited
	4-(Methylnitrosamino)-1-(3-pyridyl)-1- butanone (NNAL)	1	limited

### Analysis of urinary biomarkers in smokers and e-cigarette users

#### Polycyclic aromatic hydrocarbons

**Figure 2:**
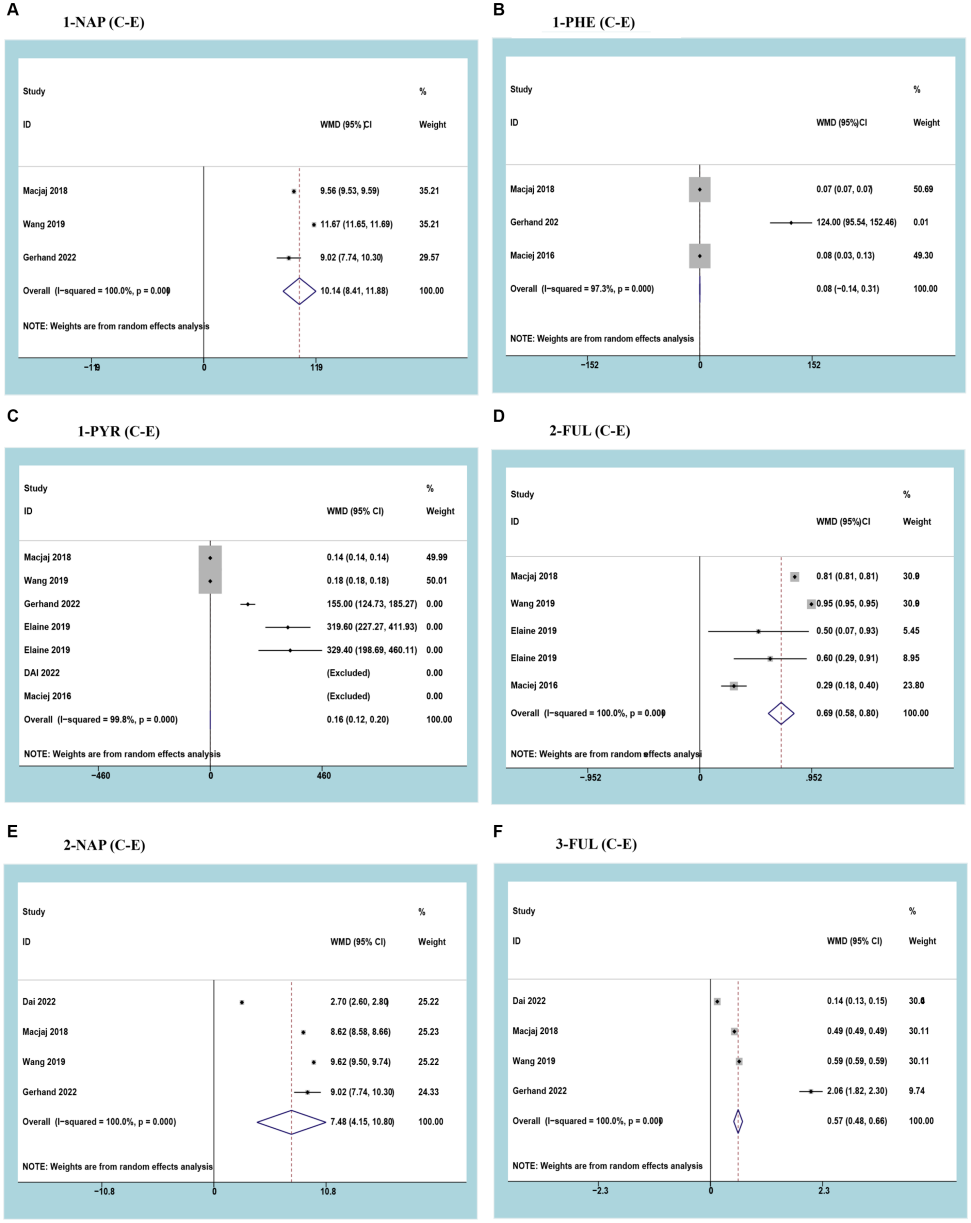
Forest plot and meta-analysis of the relationship between **(A)** 1-NAP, **(B)** 1-PHE, **(C)** 1-PYR, **(D)** 2-FUL, **(E)** 2-NAP, and **(F)** 3-FUL in the urine of traditional tobacco users (C) and e-cigarette users (E).

Three studies reported 1-NAP with a cumulative sample of 6,922 patients. After observing significant heterogeneity between studies, a random effects model was used (*I*^2^ = 100%; *p*<0.05). The results of the meta-analysis showed that urinary 1-NAP was significantly higher in smokers than in e-cigarette users (WMD 10.14, 95% CI 8.41–11.88; *p*<0.05) (Figure 2A).

Three studies reported 1-PHE with a cumulative sample of 2,718 patients. After observing significant heterogeneity between studies, a random effects model was used (*I*^2^ = 97.3%; *p*<0.05). Meta-analysis results showed no difference in urinary 1-PHE between smokers and e-cigarette users (WMD 0.08, 95% CI -0.14-0.31; *p*>0.05) (Figure 2B).

Three studies reported 1-PYR with a cumulative sample of 9,684 patients. After observing significant heterogeneity between studies, a random effects model was used (*I*^2^ = 99.8%; *p*<0.05). The results of the meta-analysis showed that urinary 1-PYR was significantly higher in smokers than in e-cigarette users (WMD 0.16, 95% CI 0.12–0.20; *p*<0.05) (Figure 2C).

Three studies reported 2-FLU with a cumulative sample of 7,098 patients. After observing significant heterogeneity between studies, a random effects model was used (*I*^2^ = 100%; *p*<0.05). The results of the meta-analysis showed that urinary 2-FLU was significantly higher in smokers than in e-cigarette users (WMD 0.69, 95% CI 0.58–0.80; *p*<0.05) (Figure 2D).

Three studies reported 2-NAP with a cumulative sample of 9,488 patients. After observing significant heterogeneity between studies, a random effects model was used (*I*^2^ = 100%; *p*<0.05). The results of the meta-analysis showed that urinary 2-NAP was significantly higher in smokers than in e-cigarette users (WMD 7.48, 95% CI 4.15–10.80; *p*<0.05) (Figure 2E).

Three studies reported 3-FLU with a cumulative sample of 9,684 patients. After observing significant heterogeneity between studies, a random effects model was used (*I*^2^ = 100%; *p*<0.05). The results of the meta-analysis showed that urinary 3-FLU was significantly higher in smokers than in e-cigarette users (WMD 0.57, 95% CI 0.48–0.66; *p*<0.05) (Figure 2F).

#### Volatile organic compounds

**Figure 3:**
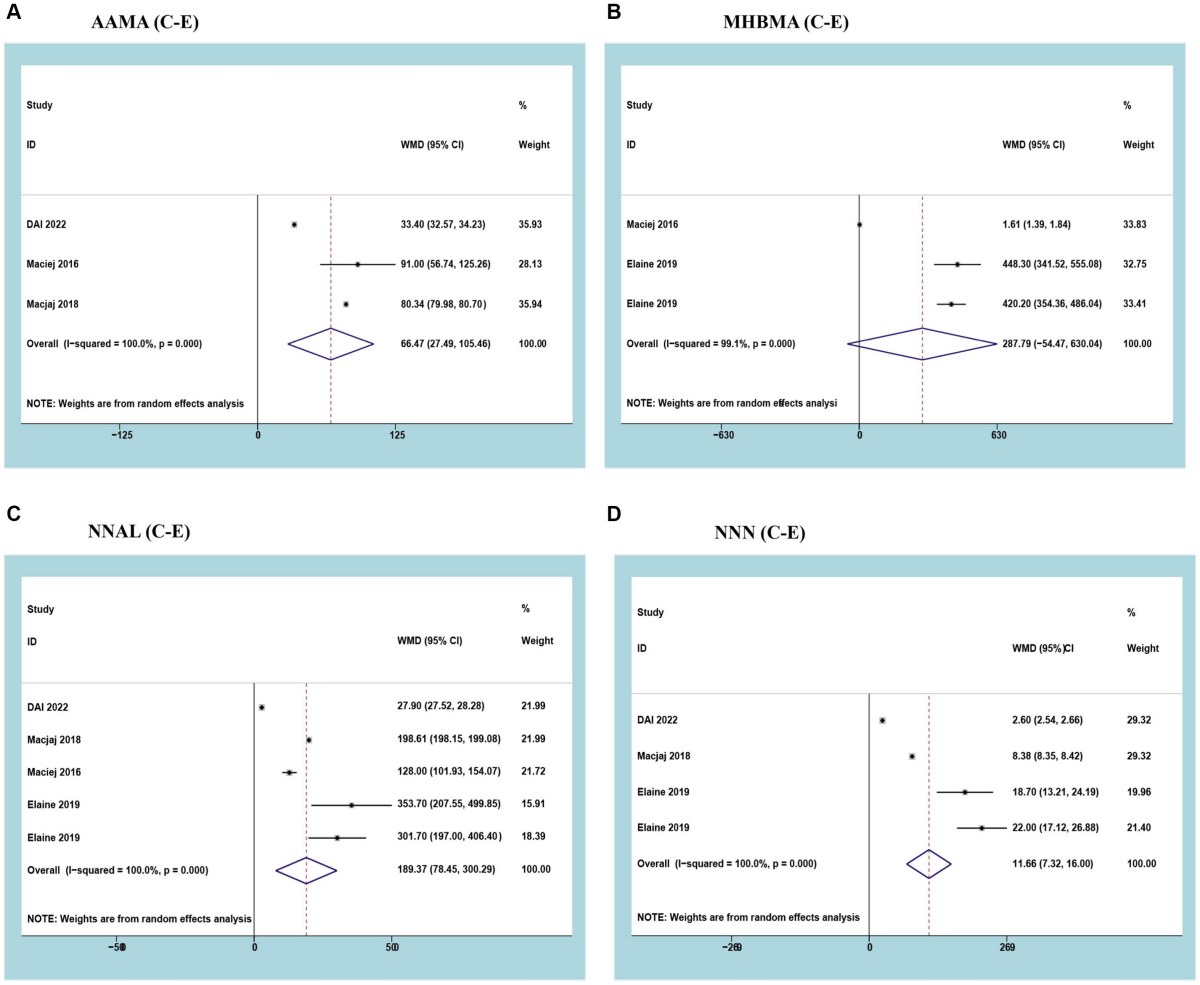
Forest plot and meta-analysis of the relationship between **(A)** AAMA, **(B)** MHBMA, **(C)** NNAL, and **(D)** NNN in the urine of traditional tobacco users (C) and e-cigarette users (E).

Three studies reported AAMA with a cumulative sample of 5,264 patients. After observing significant heterogeneity between studies, a random effects model was used (*I*^2^ = 100%; *p*<0.05). The results of the meta-analysis showed that urinary AAMA was significantly higher in smokers than in e-cigarette users (WMD 66.47, 95% CI 27.49–105.46; *p*<0.05) (Figure 3A).

Three studies reported MHBMA with a cumulative sample of 196 patients. After observing significant heterogeneity between studies, a random effects model was used (*I*^2^ = 99.1%; *p*<0.05). Meta-analysis results showed no difference in urinary MHBMA between smokers and e-cigarette users (WMD 287.79, 95% CI -54.47-630.04; *p*>0.05) (Figure 3B).

Three studies reported NNAL with a cumulative sample of 5,420 patients. After observing significant heterogeneity between studies, a random effects model was used (*I*^2^ = 100%; *p*<0.05). The results of the meta-analysis showed that urinary NNAL was significantly higher in smokers than in e-cigarette users (WMD 189.37, 95% CI 78.45–300.29; *p*<0.05) (Figure 3C).

Three studies reported NNN with a cumulative sample of 5,380 patients. After observing significant heterogeneity between studies, a random effects model was used (*I*^2^ = 100%; *p*<0.05). The results of the meta-analysis showed that urinary NNN was significantly higher in smokers than in e-cigarette users (WMD 11.66, 95% CI 7.32–16.00; *p*<0.05) (Figure 3D).

### Subgroup analysis

Urine biomarker levels were analyzed in nonsmokers and traditional tobacco users, nonsmokers and e-cigarette users, nonsmokers and dual users, traditional tobacco users and dual users, and e-cigarette users and dual users. The analysis revealed that urinary biomarkers were significantly higher in traditional tobacco users than in non-smokers, in e-cigarette users than in non-smokers, and in dual users than in non-smokers, traditional tobacco users, and e-cigarette users (Supplementary Figure S1).

### Sensitivity analysis

**Figure 4:**
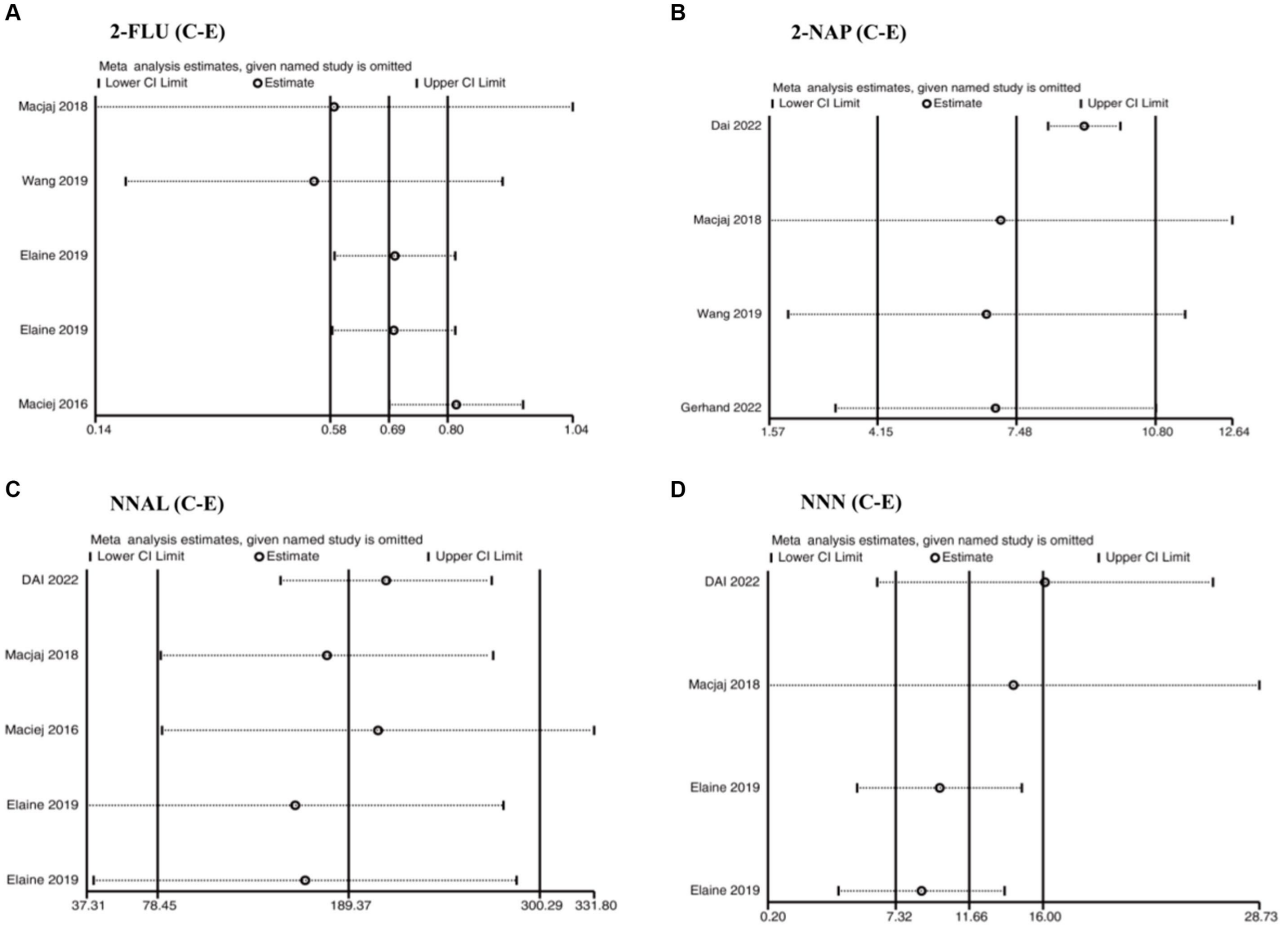
Sensitivity analysis of the relationship between **(A)** 2-FUL, **(B)** 2-NAP, **(C)** NNAL, and **(D)** NNN in the urine of traditional tobacco users (C) and e-cigarette users (E).

High heterogeneity between studies could not be avoided, despite the fact that all included studies received high quality scores (at least six stars) after a rigorous assessment of the quality of the literature. Sensitivity analyses were performed to track the heterogeneity of each outcome metric. In the cases of outcomes with high heterogeneity (EBL and LOS), the included studies were individually excluded so that statistical merging and heterogeneity tests could be performed again to clarify the changes. This step was followed by STATA (Figure 4). Sensitivity analyses were also conducted for other subgroups to track the heterogeneity of each outcome indicator (Supplementary Figure S2).

### Publication bias

Begg’s test was performed and no publication bias was detected in the studies included in the analysis (*p*>0.05) (Table 4) (Supplementary Figure S3).

**Table 4:** Publication bias analysis of the relationship between Traditional tobacco users (C) and E-cigarette users (E).

Urinary biomarker(s) (abbreviation)	Number of studies	*t*	*p*	95% Confidence interval	
1-PYR	6	0.22	0.843	−50.19608	57.52009
2-NAP	4	−0.42	0.713	−297.1512	243.8456
3-FUL	4	−0.54	0.641	−411.2353	318.8682
2-FUL	4	−0.04	0.974	−107.1313	104.7418
1-PHE	3	1.11	0.468	−47.35785	56.3814
1-NAP	3	−0.19	0.880	−1351.402	1311.359
AAMA	3	−0.52	0.697	−1076.371	992.3335
MHBMA	3	4.83	0.130	−16.8817	37.59594
NNN	4	−0.24	0.830	−379.1978	338.4354
NNAL	5	0.17	0.877	−559.1644	621.6045

## Discussion

The impact of traditional cigarettes in comparison to e-cigarettes on bladder cancer has not been explored in an analogous published study. Existing e-cigarette research predominantly centers on the documentation of urinary biomarkers pertaining to three chemical groups: PAHs, VOCs, and TANAs. These chemical categories have undergone extensive investigation in both combustible tobacco and e-cigarette usage, with a suite of well-established analytical techniques in place for characterizing the carcinogens present in urine (17). Therefore, we implemented this meta-analysis, covering a large sample of 16,876 patients, to fill this research gap. The meta-analysis included data from 10 controlled studies that helped compare carcinogens associated with bladder cancer in the urine of traditional tobacco users and e-cigarette users. The results of the study clearly showed that traditional tobacco users had significantly higher levels of bladder carcinogens (including PAHs, VOCs and TSNs) in their urine than e-cigarette users.

PAHs are a class of organic compounds that contain multiple benzene rings, and are mainly produced by the combustion of organic substances, automobile exhaust, and industrial emissions. Significant sources of human exposure to PAHs are the production and use of coal-derived products, tobacco smoke and air (International Agency for Research of cancer (IARC)) (18). The carcinogenic properties of PAHs have garnered significant scientific attention. Numerous studies indicate a correlation between elevated concentrations of PAHs and cancer development. The two primary aspects are as follows: Direct carcinogenicity, where certain substances within PAHs are thought to exert direct carcinogenic effects on humans by interacting with DNA, causing genetic mutations, and thereby increasing cancer risk. For example, PAHs such as 1-NAP, 2-NAP, 1-PHE, 1-PYR, 2-FLU and 3-FLU are considered to be directly carcinogenic (19, 20). Indirect carcinogenicity involves the activation of specific metabolic processes by PAHs, leading to cellular and tissue damage and subsequently elevating the risk of cancer. The presence of these chemicals in urine is believed to be carcinogenic to the epithelium of the bladder urinary tract (21). In order to better compare the exposure to PAHs in the urine of conventional tobacco and e-cigarettes, we counted the carcinogenic markers in the urine of tobacco users and e-cigarette users in six articles (11–16). A weighted analysis of non-smokers versus traditional tobacco users, e-cigarette users, and dual users showed that traditional tobacco users had significantly more carcinogens in their urine than e-cigarette users and negligible environmental PHA compared to smoke and e-cigarette production. In the sensitivity analyses, we noted a large effect on heterogeneity in a study by Elaine et al. The reason for this was that they did not use a standard formula test, but rather collected 24-h urine specimens for the test, thus increasing the exposure to the urinary environment leading to increased heterogeneity. Interesting findings were that there was no significant difference in urinary 1-PHE between traditional tobacco users and e-cigarette users, and that it was much higher than in the urine of non-smokers. This suggests that although e-cigarettes are a safer alternative to traditional tobacco, they are still potentially dangerous.

VOCs are a class of organic compounds with high vapor pressure at room temperature that can evaporate into the air. The International Agency for IARC classifies VOCs differently, and AAMA and MHBHA in our meta-analysis are strongly carcinogenic, but the mechanism of their effects on bladder cancer is not known (22, 23). However, since the urinary levels were significantly higher in traditional cigarette and e-cigarette users than in nonsmokers, it is believed that AAMA and MHBMA have some effect on bladder uroepithelial cells. A meta-analysis of the four articles showed that AAMA and MHBMAHA in the urine of tobacco users were significantly higher than those of e-cigarette users, even when environmental influences such as secondhand smoke could not be excluded (11–14).

TSNAs, a subgroup of nitrosamines, are chemical compounds produced in the growth, processing, and preparation of tobacco, rendering them carcinogenic. Formed through the reaction of nicotine with nitrites, TSNAs are prevalent in smoking-related products such as cigarettes, cigars, and smokeless tobacco. This metabolic process results in the formation of reactive intermediates that induce damage to DNA and other cellular components (24). Elaine et al.’s randomized, parallel-group clinical study recruited smokers to switch to different flavored e-cigarettes or Nicorette 4 mg nicotine gum in a controlled environment. MHBMA, NNN and NNAL were found to be significantly lower in those who switched to e-cigarettes than in traditional tobacco users. This experiment is a good indication that e-cigarettes are an effective alternative to traditional tobacco. However, it is necessary to strengthen the regulation and management of e-cigarettes as adolescents only like the taste of e-cigarettes containing flavors rather than using e-cigarettes as substitutes for traditional tobacco, but the chemicals produced by e-cigarettes can cause harm to adolescents.

Remarkably, our study determined that despite considerable environmental influences, the analyzed results consistently position e-cigarettes as a safer alternative to traditional tobacco. The significantly higher levels of carcinogens in the urine of e-cigarette users compared to non-smokers suggests the need to strengthen protective measures for minors.

### Limitations

While the meta-analysis adhered to the PRISMA guidelines, certain limitations are inherent. Firstly, not all included studies conformed to the rigorous standards of high-quality randomized controlled trials, resulting in a somewhat compromised level of evidence. Secondly, the geographic scope of the included studies was relatively narrow, exclusively from the United States, predominantly featuring Caucasian ethnicity and spanning a broad age range, thus introducing selection bias. Thirdly, the extensive dataset prevented the differentiation between nonsmokers influenced by secondhand smoke or environmental biomarkers.

## Conclusion

Evidence from several studies has shown that bladder cancer carcinogens in the urine of e-cigarette users are significantly lower than those of traditional tobacco users, but bladder cancer carcinogens in the urine of e-cigarette users are significantly higher than those of non-smokers. This suggests that e-cigarettes, although a safer alternative to traditional tobacco, are still potentially harmful. However, the long-term effects of prolonged exposure of the urinary tract epithelium of e-cigarette users to urinary tract carcinogens are unknown, but the presence of bladder cancer carcinogens in urine after using e-cigarettes should be of sufficient concern.

## Data availability statement

The original contributions presented in the study are included in the article/Supplementary materials, further inquiries can be directed to the corresponding authors.

## Author contributions

LF: Data curation, Formal analysis, Writing – review & editing, Software, Writing – original draft. GH: Data curation, Methodology, Formal analysis, Writing – review & editing. LP: Formal analysis, Software, Writing – original draft, Writing – review & editing, Conceptualization, Funding acquisition, Methodology, Validation. RL: Conceptualization, Formal analysis, Writing – review & editing, Investigation, Supervision, Visualization. DD: Conceptualization, Visualization, Methodology, Project administration, Resources, Software, Validation, Writing – original draft. SZ: Conceptualization, Methodology, Software, Validation, Visualization, Investigation, Writing – review & editing. GL: Conceptualization, Investigation, Methodology, Software, Visualization, Formal analysis, Funding acquisition, Resources, Writing – original draft, Writing – review & editing. SW: Formal analysis, Funding acquisition, Investigation, Writing – review & editing, Data curation.
