# Supplementary material for: Comparison of bladder carcinogenesis biomarkers in the urine of traditional cigarette users and e-cigarette users
Source: Front Public Health. 2024 Apr 23;12:1385628. doi: 10.3389/fpubh.2024.1385628 (PMC11075070; doi:10.3389/fpubh.2024.1385628)

Supplementary Material

**Figure S1.** **Forest plot and meta-analysis of the relationship between different subgroups of non-smokers (N), traditional tobacco users (C), e-cigarette users (E) and dual users (D)**

**
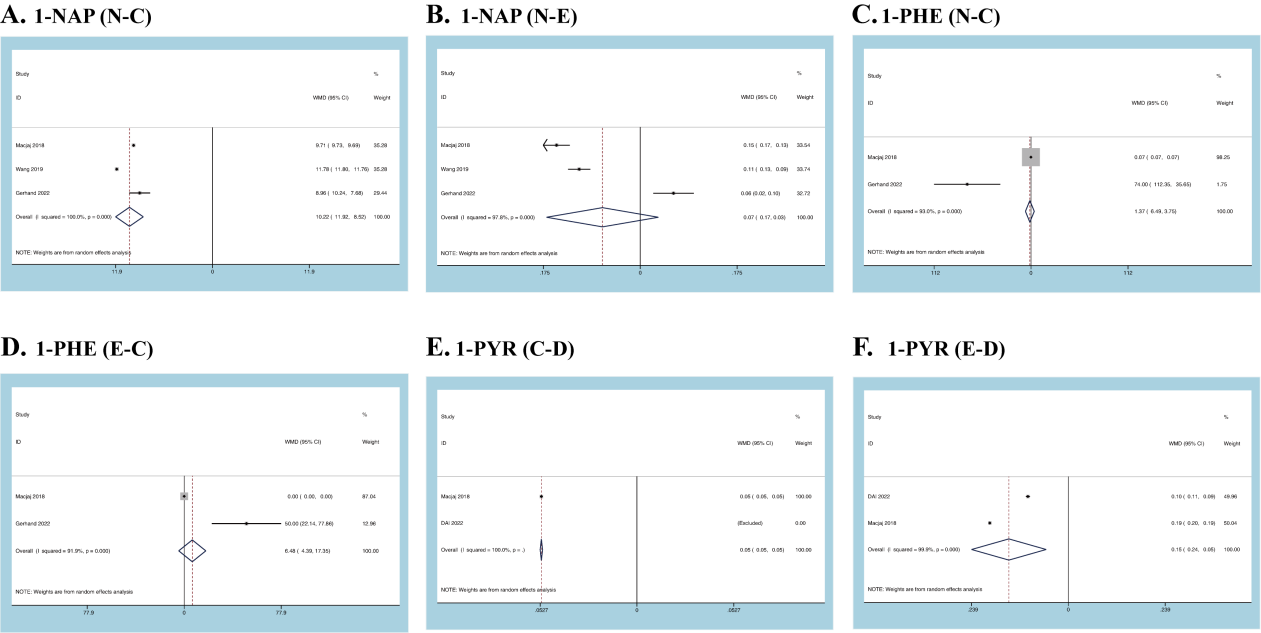

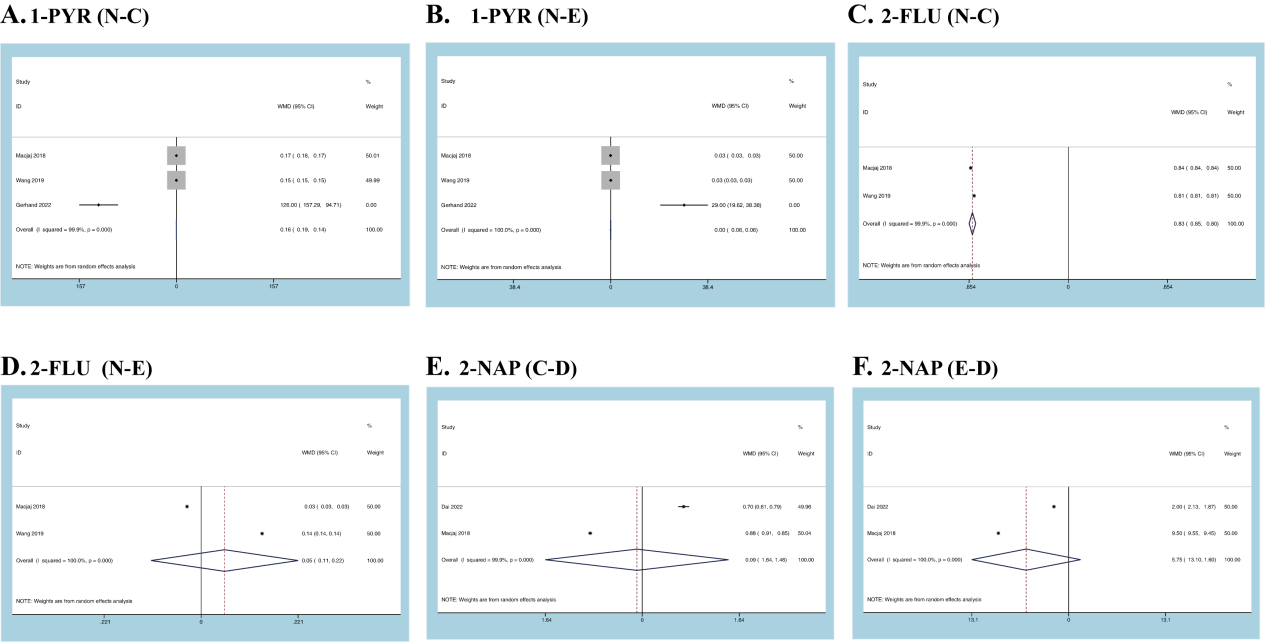

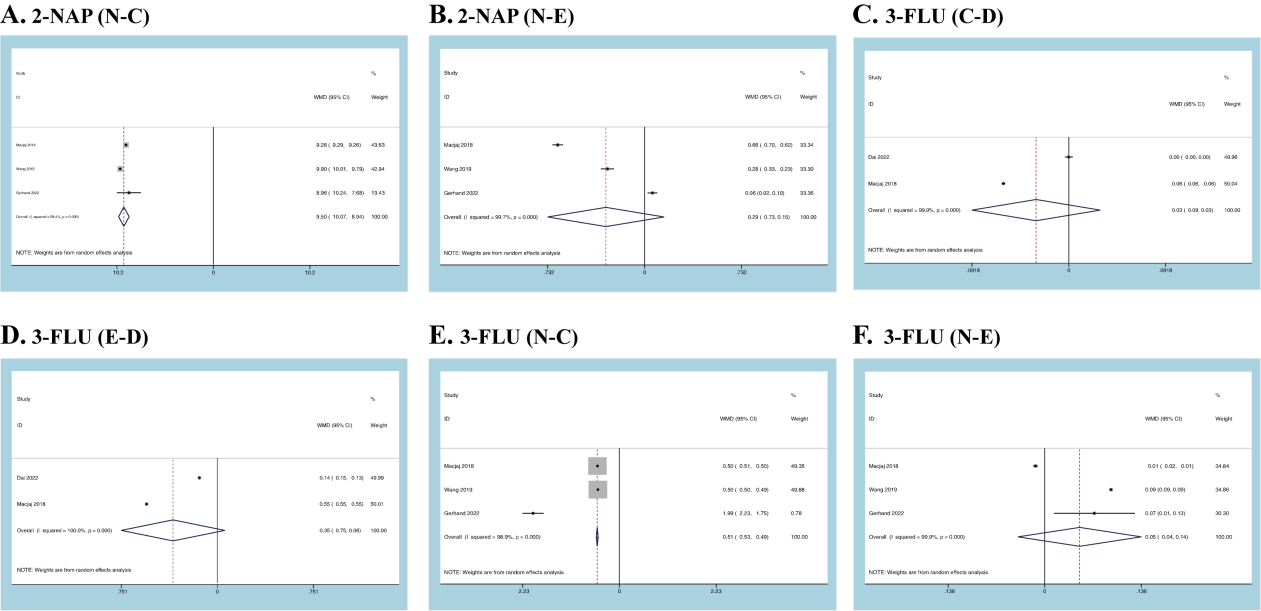

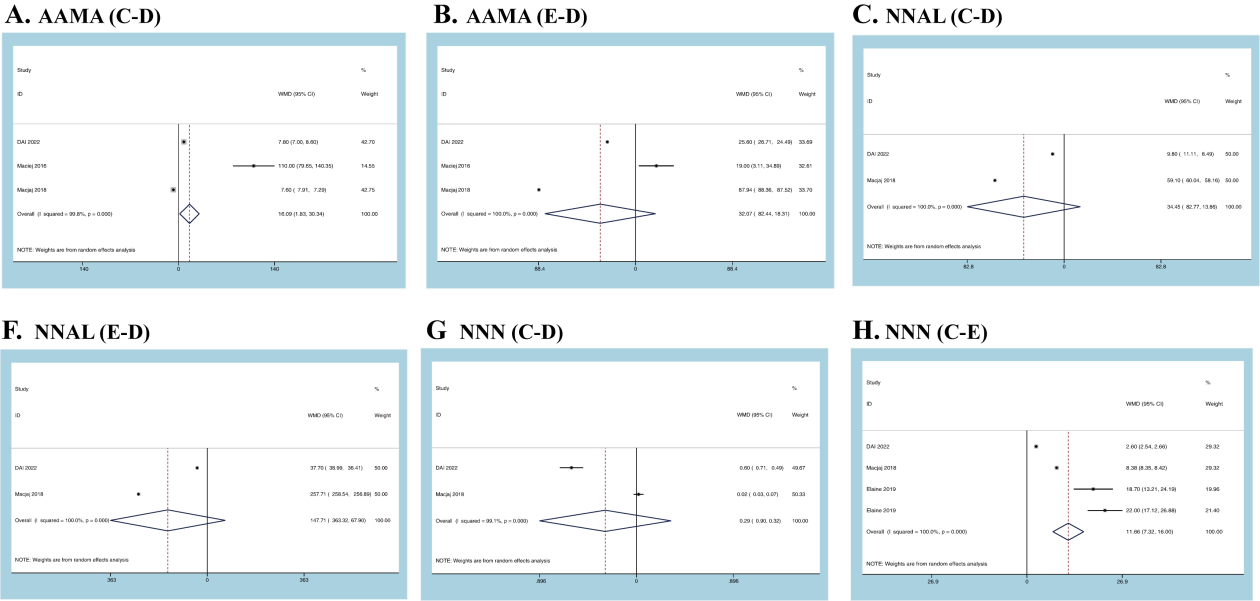
**

**Figure S2. Sensitivity analysis of the relationship between different subgroups of non-smokers (N), traditional tobacco users (C), e-cigarette users (E) and dual users (D)**


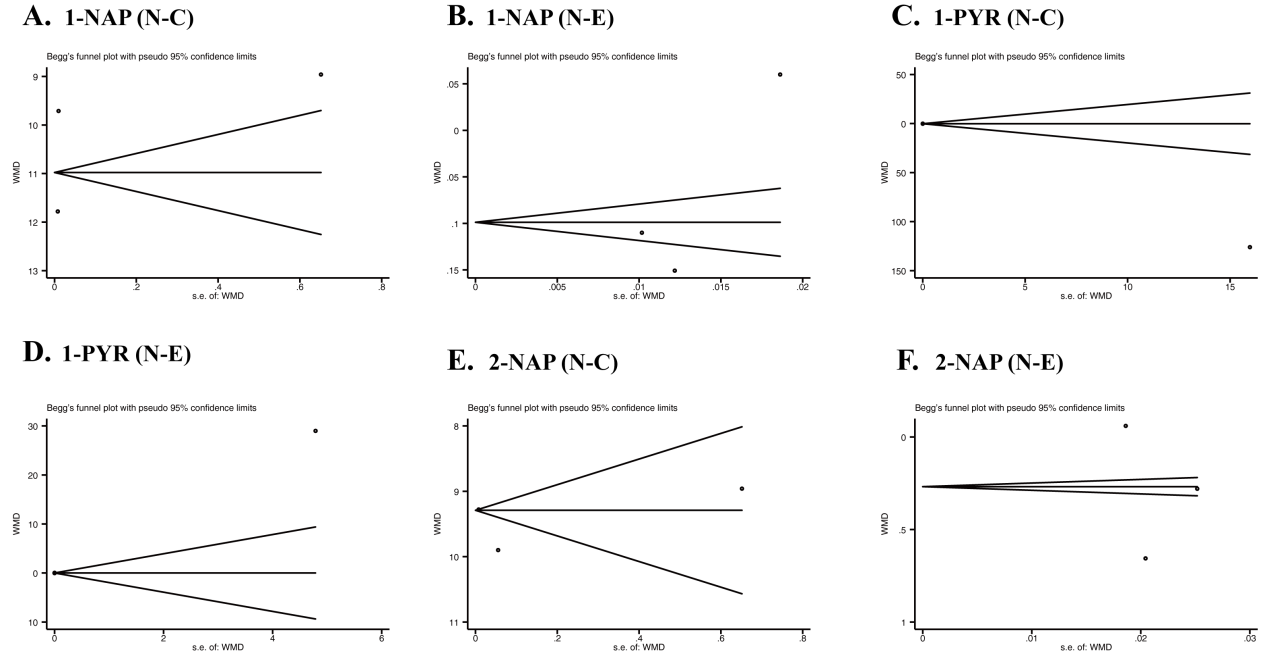

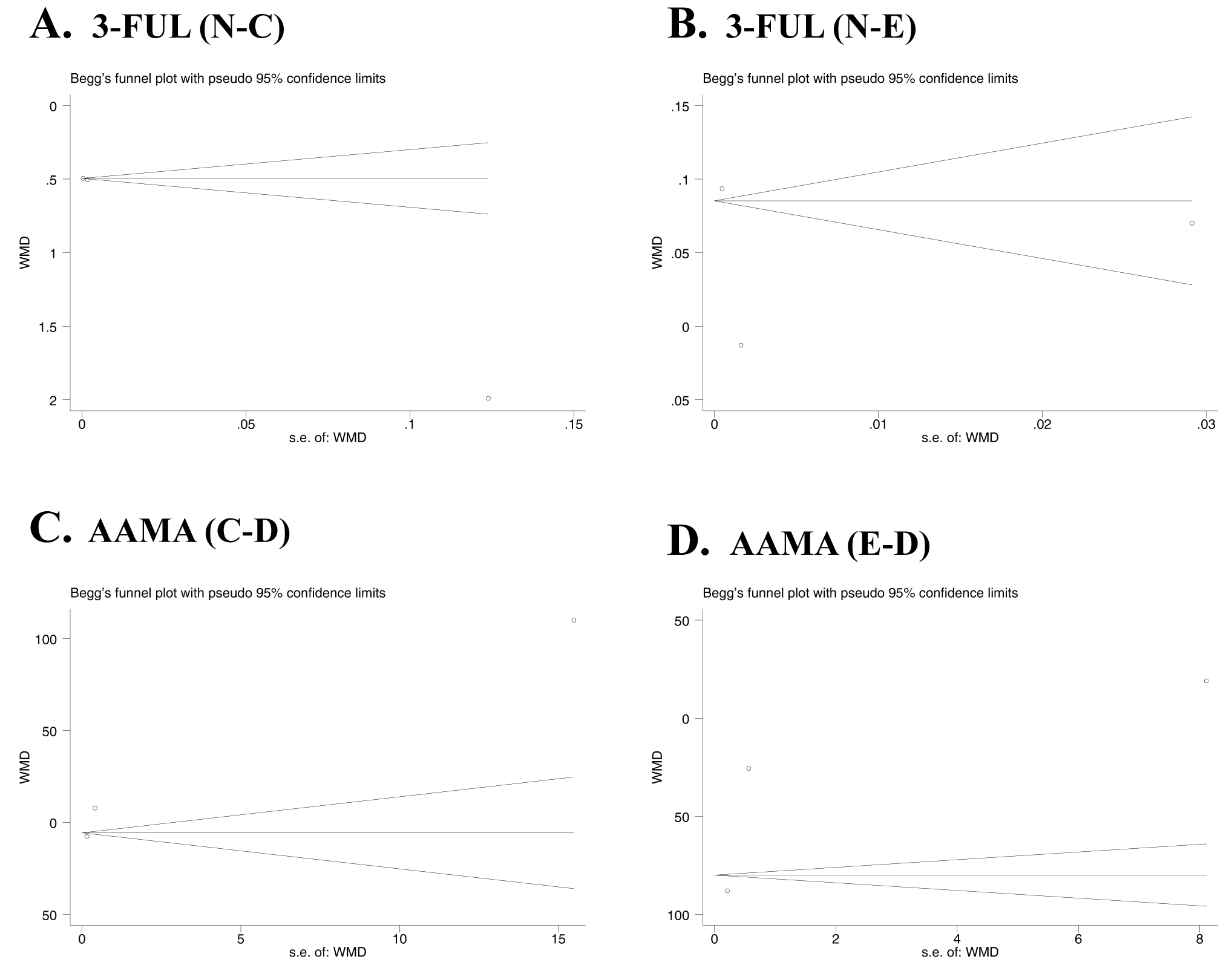


**Figure S3. Publication bias analysis of the relationship between different subgroups of non-smokers (N), traditional tobacco users (C), e-cigarette users (E) and dual users (D)**


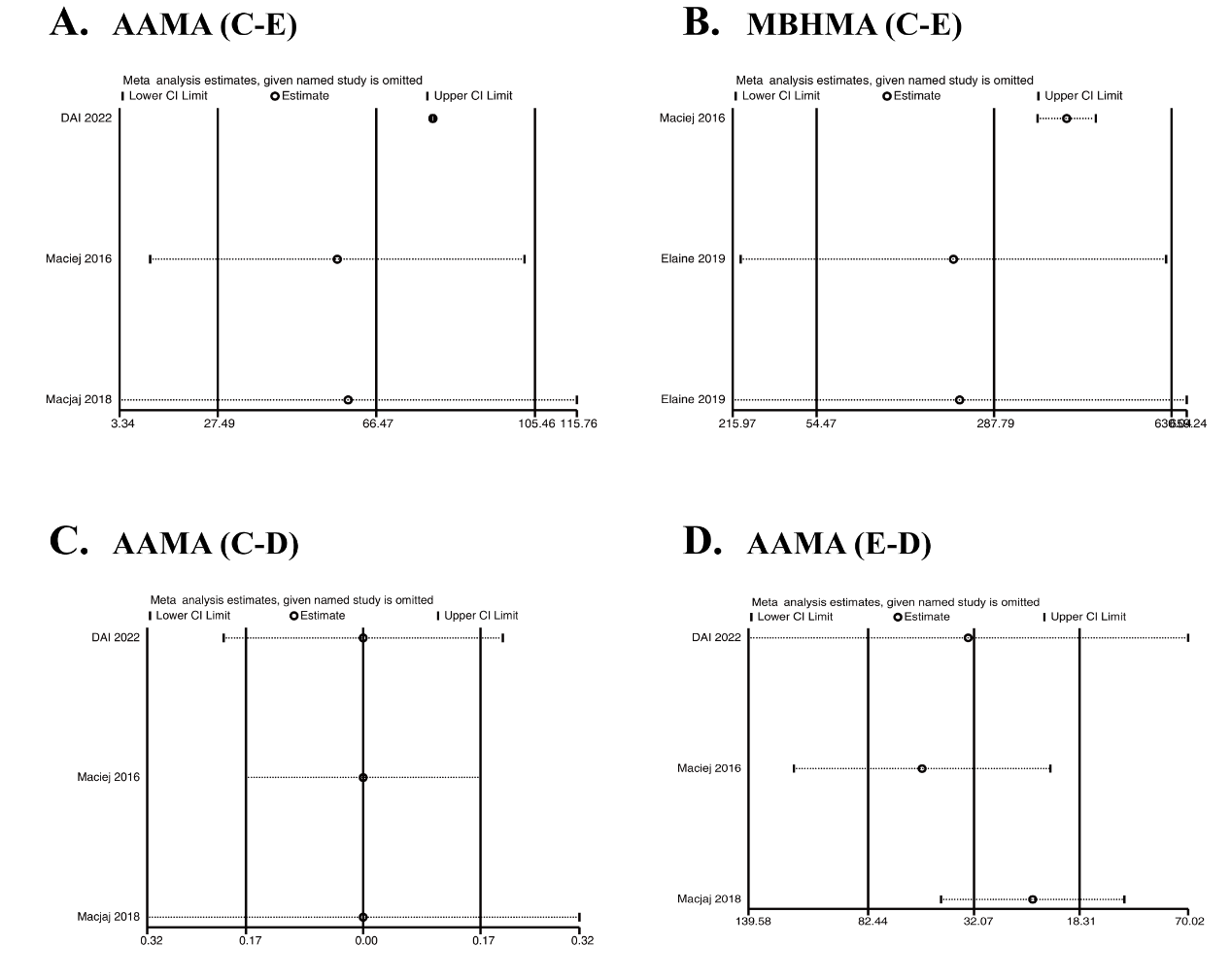

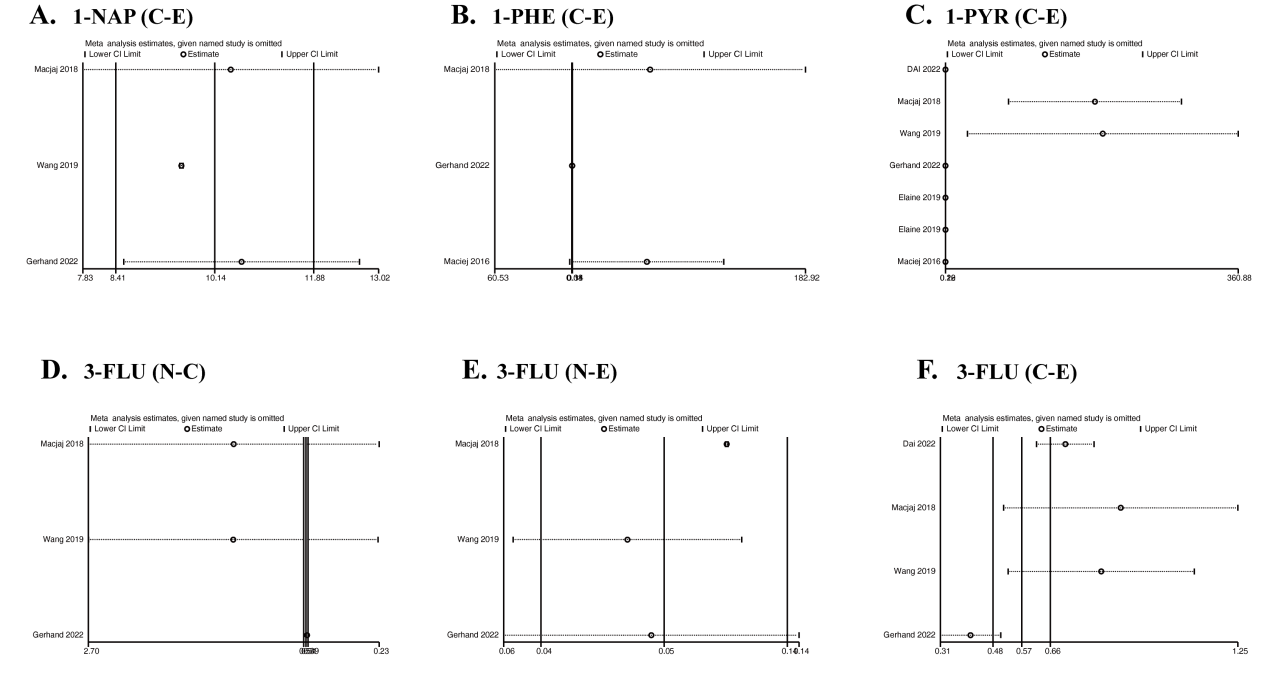


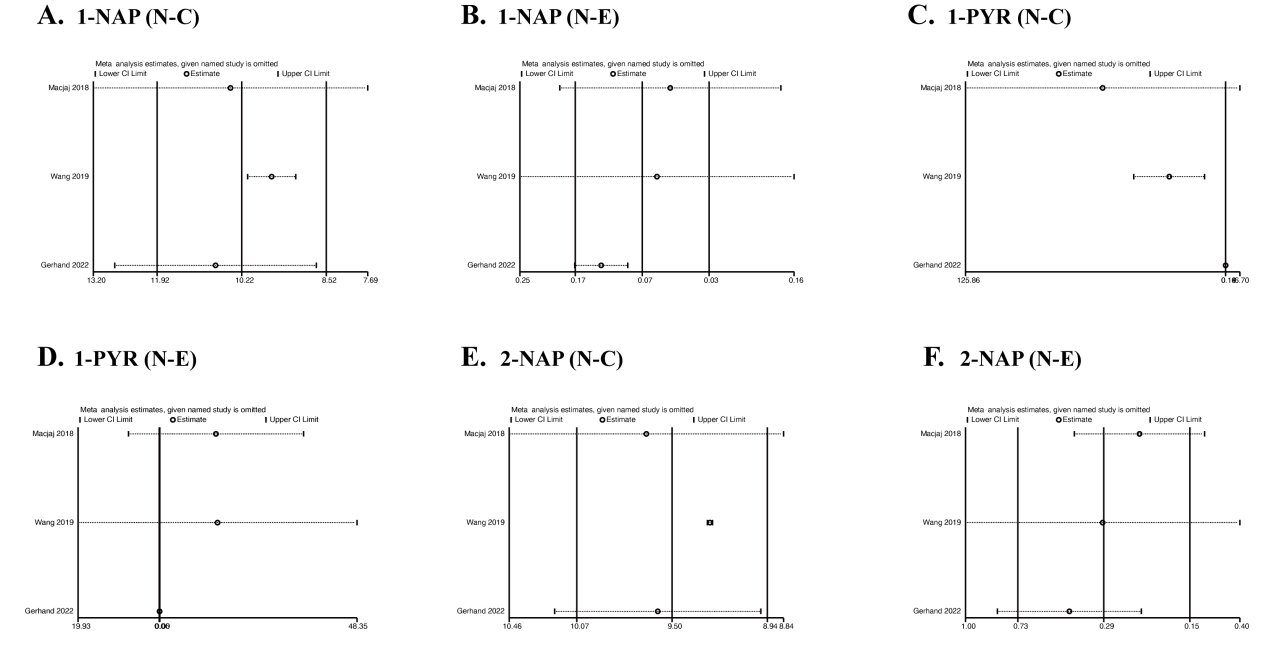

Supplement: Supplementary file 1 [file Data_Sheet_1.docx]
